# Over-expression of Adenine Nucleotide Translocase 1 (ANT1) Induces Apoptosis and Tumor Regression *in vivo*

**DOI:** 10.1186/1471-2407-8-160

**Published:** 2008-06-04

**Authors:** Ji-Young Jang, Yun Choi, Yoon-Kyung Jeon, Khin Chaw Yu Aung, Chul-Woo Kim

**Affiliations:** 1 Department of Pathology, Tumor Immunity Medical Research Center, Cancer Research Institute, Seoul National University College of Medicine, 28 Yeongeon-dong, Jongno-gu, Seoul 110-799, South Korea

## Abstract

**Background:**

Adenine nucleotide translocase (ANT) is located in the inner mitochondrial membrane and catalyzes the exchange of mitochondrial ATP for cytosolic ADP. ANT has been known to be a major component of the permeability transition pore complex of mitochondria and contributes to mitochondria-mediated apoptosis. Human ANT has four isoforms (ANT1, ANT2, ANT3, and ANT4), and the expression of the ANT isoforms is variable depending on the tissue and cell type, developmental stage, and proliferation status. Among the isoforms, ANT1 is highly expressed in terminally-differentiated tissues, but expressed in low levels in proliferating cells, such as cancer cells. In particular, over-expression of ANT1 induces apoptosis in cultured tumor cells.

**Methods:**

We applied an ANT1 gene transfer approach to induce apoptosis and to evaluate the anti-tumor effect of ANT1 in a nude mouse model.

**Results:**

We demonstrated that ANT1 transfection induced apoptosis of MDA-MB-231 cells, inactivated NF-κB activity, and increased Bax expression. ANT1-inducing apoptosis was accompanied by the disruption of mitochondrial membrane potential, cytochrome c release and the activation of caspases-9 and -3. Moreover, ANT1 transfection significantly suppressed tumor growth *in vivo*.

**Conclusion:**

Our results suggest that ANT1 transfection may be a useful therapeutic modality for the treatment of cancer.

## Background

Apoptosis is a programmed form of cell death, and as such, differs fundamentally from cellular necrosis. Apoptosis is characterized by genetically-controlled cellular auto-digestion via the activation of endogenous proteases [1]. Apoptosis results in cytoskeletal disruption, cell shrinkage, membrane blebbing, nuclear condensation, and inter-nucleosomal DNA fragmentation [1,2]. Moreover, apoptosis is essentially required for the homeostasis of normal tissues and to manage cellular disruption caused by mutations. Hence, the disruption of the apoptotic process is involved in the pathogenesis of many human diseases, including viral infections, autoimmune diseases, and cancers. In particular, progressive perturbations of the normal apoptotic pathway occur during neoplastic transformation, progression, and metastasis [3], and thus apoptosis-related genes are attractive cancer treatment developmental targets [4].

Adenine nucleotide translocase (ANT) participates in ATP-for-ADP exchange through the inner mitochondrial membrane, which supplies the cytoplasm with newly synthesized ATP for oxidative phosphorylation [5]. Recently, it has been reported that death receptor-initiated pathway, mediated by RIP, disrupts the interaction between cyclophilin D and ANT and permits the binding of zVAD.fmk (zVAD) to ANT, which prevents ANT from adopting the conformational c-state and subsequently results in inhibition of ADP/ATP exchange, the reduction of cellular ATP, and necrotic cell death [6].

Four closely related isoforms of ANT (ANT1, 2, 3, and 4) exist in humans, and these are expressed in a tissue-specific manner [7]. ANT1 is predominantly expressed in the heart, skeletal muscle, and brain; ANT2 is predominantly expressed in the liver and in cells with increased proliferative activity; and ANT3 is ubiquitously detected [8]. ANT1 was first identified as an apoptosis-inducing protein. ANT1 over-expression induces rapid cell death with a concomitant decrease in Δψm and an increase in nucleosomal DNA degradation [9,10]. However, in the majority of cancer cell lines, ANT1 expression is minimal, whereas the expression of ANT2, an alleged anti-apoptotic molecule, is high [11]. In cancer cell lines, up-regulation of ANT1 expression resulted in the recruitment of an I-κBα-NF-κB-complex into mitochondria and a concomitant decrease in nuclear factor kappa B (NF-κB) binding activity [12]. Increasing evidences suggest that NF-κB plays an important role in tumor development and progression [13]. Moreover, constitutive NF-κB activation due to signaling defects, mutations, or chromosomal rearrangements has been identified in a wide variety of cancers [13,14], and thus the constitutive activation of NF-κB is viewed as an obstacle to effective cancer therapy [15].

We hypothesized that ANT1 gene transfer into cancer cells would inhibit cell growth and induce apoptosis through NF-κB inactivation, and investigated the anti-cancer effect of ANT1 over-expression *in vitro *and *in vivo *using a nude mouse model.

## Methods

### Cell line and culture

MDA-MB-231 cells were used throughout this study and were purchased from the Korean Cell Line Bank (Seoul, Korea). MDA-MB-231 cells were cultured in DMEM medium supplemented with 10% fetal bovine serum (FBS), 100 units/ml penicillin, and 100 μg/ml streptomycin in a humidified 5% CO_2_/95% air atmosphere at 37°C.

### Reverse transcription-PCR (RT-PCR)

Cancer cell lines were collected and total RNA was extracted using Trizol (Invitrogen, Carlsbad, CA, USA), according to the manufacturer's instructions. For reverse transcription-polymerase chain reaction (RT-PCR) analysis, 5 μg of total RNA was reverse-transcribed using RT-PCR kits (Promega, Madison, WI, USA). PCR was used to amplify target cDNA under the following conditions: 35 cycles at 94°C for 1 minute, 55°C for 1 minute, and 72°C for 2 minutes. PCR products were analyzed using standard agarose gel electrophoresis. The primers used for RT-PCR were as follows; ANT1 forward 5'-ACAGATTGTGTGGTTT-3' and reverse 5'-TTTTGTGCATTAAGTGGTCTTT-3, and β-actin forward 5'-GGAAATCGTGCGTGACA TTAAGG-3' and reverse 5'-GGCTTTTAGGATGGCAAGGGAC-3'.

### Transfection

ANT1 expression vector (pcDNA 3.1-ANT1) was kindly provided by Dr. S. Grim (Max-Planck Institute, Berlin, Germany). To transfect MDA-MB-231 cells with plasmid vector, cells were plated into either 6-well plates (2 × 10^5 ^cells per well) or a 100 mm dish (2 × 10^6 ^cells) and allowed to adhere for 24 hours. Lipofectamine 2000 (Invitrogen) was used for the transfection, and after pcDNA3.1 or pcDNA3.1-ANT1 transfection, cells were cultured for 4 hours and then the medium was replaced with fresh medium supplemented with 10% fetal bovine serum. Cells were harvested 24–48 hours after transfection.

### FACS cytometric analysis of apoptotic cells

Approximately 2 × 10^5^/ml MDA-MB-231 cells were transfected with ANT1 and cultured for the indicated length of time. Cells were then harvested, washed twice with PBS, and incubated with reagent containing Annexin V conjugated with fluorescence isothiocyanate (FITC; 2.5 μg/ml) and propidium iodide (PI, 5 μg/ml; BD Pharmingen, San Diego, CA, USA) for 15 min at room temperature. Cells were analyzed using a flow cytometer (Epics XL; Coulter, Marseille, France).

### Measurement of mitochondrial membrane potentials (Δψ_m_)

To measure levels of Δψ_m _disruption, cells were harvested, washed twice with PBS, and then incubated with 20 nM 3,3'-diethyloxacarbocyanine (DiOC_6_; Molecular Probes, Eugene, OR, USA) for 15 min at 37°C. Δψ_m _values were determined by a flow cytometer (Epix XL).

### DNA fragmentation assays

Cells were subjected to DNA fragmentation analysis on 2% agarose gels. Where indicated, cells were transfected with ANT1.

### Western blotting

Prepared cytosolic, nuclear, or mitochondrial lysates were analyzed for protein content using Bradford reagent (Bio-Rad, Hercules, CA, USA). Briefly, 50 μg of total protein was electrophoresed in 10% SDS-PAGE gel, transferred to a polyvinylidene difluoride membrane (Millipore, Bedford, MA, USA), and incubated with monoclonal anti-NF-κB, anti-Bcl-xL, anti-Bax, anti-cyt c, anti α-tubulin (Santa Cruz Biotechnology, Santa Cruz, CA, USA), anti-caspase-9 (BD Pharmingen), anti-caspase-3 (Stressgen, Victoria, Canada), anti-phospho-Akt, or anti-Akt antibodies (Cell Signaling Technology, Berverly, MA, USA). Immunoblots were visualized by enhanced chemiluminescence (Amersham Pharmacia Biotech, Uppsala, Sweden).

### Confocal microscopy

Cells were grown in Lab-tek chamber slides (177429; NUNC, Naperville, IL, USA). After fixing cells for 10 minutes at room temperature, cells were washed four times with filtered PBS and blocked with 3% fetal bovine serum in 1% BSA/PBS for 30 minutes at room temperature. Cells were then washed three times with filtered PBS and incubated overnight at 4°C with monoclonal anti-NF-κB primary antibodies (Santa Cruz Biotechnology). After three washes, cells were incubated with FITC-conjugated secondary antibodies or DAPI for 30 minutes at room temperature. Images were obtained using a LSM510 unit (Carl Zeiss, Jena, Germany).

### NF-κB reporter gene assay

The NF-κB-luciferase-reporter construct containing four tandem NF-κB signal binding motifs (Clontech, Palo Alto, CA, USA) was co-transfected with pcDNA3.1 or pcDNA3.1-ANT1 into MDA-MB-231 cells using Lipofectamine 2000 (Invitrogen), and cultured for the indicated times. Culture media were then removed, cells were rinsed gently with PBS, and 200 μl of 1 × lysis buffer was added per well. Cells were then incubated with 1 × lysis buffer at 4°C for 15–20 minutes and one freeze-thaw cycle was also performed to achieve complete lysis. Cell lysates were then transferred to pre-labeled microcentrifuge tubes and centrifuged at 12,000 g for 4 minutes at 4°C. Supernatant fractions (cell extracts) were recovered and luciferase activities were determined using a single-sample luminometer (FB12 luminometer; Berthold Detection Systems, Pforzheim, Germany).

### In vivo study

A tumor model was established in 6- to 8-week-old Balb/c nude mice by subcutaneous injection of 5 × 10^6 ^MDA-MB-231 cells. Therapy was started 2 weeks after tumor inoculation, when the tumor volume reached approximately 100 mm^3^. pcDNA3.1 (as a vector controls) or pcDNA3.1-ANT1 (100 μg) with Lipofectamine 2000 (100 μl) were administrated by intratumoral injection twice per day on post-challenge day 15 and 16. Tumor size was measured with a caliper every 3 or 7 days for 40 days, and tumor volumes were calculated using *m*_1_^2 ^× *m*_2 _× 0.5236 (where *m*_1 _represents tumor short axis and *m*_2 _long axis) [16].

### Statistical analysis

Data were analyzed using Student's *t *test, and a *p *value of < 0.05 was considered to be statistically significant in the experiments.

## Results

### 1. ANT1 expression was low in various human cancer cell linesand the over-expression of ANT1 by transfection of pcDNA 3.1-ANT1 induced apoptotic cell death *in vitro*

**Figure 1:**
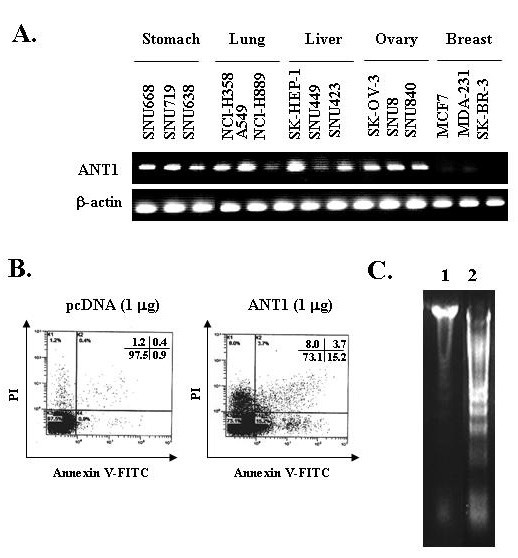
**Expression of ANT1 in various human cancer cells and the effect of ANT1 expression vector in human breast cancer cells**. A. To evaluate the levels of ANT1 expression levels in human cancer cell lines from various origins (SNU668, SNU719, SNU638, NCI-H358, A549, NCI-H889, SK-HEP-1, SNU449, SNU423, SK-OV-3, SNU8, SNU840, MCF7, MDA-MB-231, and SK-BR-3), total RNA was extracted and subjected to RT-PCR using specific primers for human ANT1 or β-actin as an internal control. B. Cells were transfected with pcDNA-ANT1; 24 hours later, the cells were stained with Annexin V-FITC and PI, and cell death was analyzed by flow cytometry. C. DNA fragmentation was nduceded by ANT1 over-expression Cells were transfected with pcDNA-ANT1 and 24 hours later, the total genomic DNA was extracted and DNA fragmentation was analyzed by 2% agarose gel electrophoresis.

We first determined the expression levels of ANT1 in variable human cancer cell lines to investigate the availability of ANT1 as a target for cancer gene therapy. ANT1 expression measured by RT-PCR was very low in many human cancer cells examined (Fig. 1A). Especially, ANT1 was scarcely detected in human breast cancer cell lines, including MCF7, MDA-MB-231, and SK-BR-3. Based on these results, human breast cancer cell lines were used throughout further study. Over-expression of ANT1 in MDA-MB-231 cells through transfection of pcDNA3.1-ANT1 vector induced cell death by 30–40% after 24 hours as determined by Annexin V-PI staining and FACS analysis (Fig. 1B). DNA laddering was also observed in MDA-MB-231 cells transfected with pcDNA3.1-ANT1 (Fig. 1C). These results demonstrated that over-expression of ANT1 by pcDNA3.1-ANT1 induced apoptotic cell death.

### 2. Over-expression of ANT1 significantly decreased NF-κB activity

**Figure 2:**
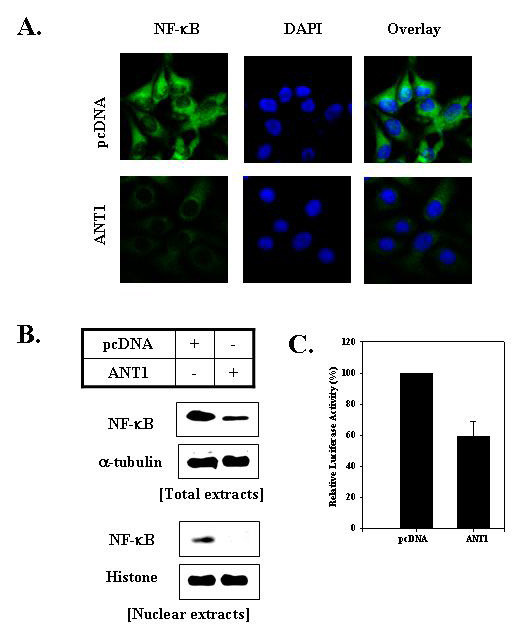
**Over-expression of ANT1 decreased the activity of NF-κB**. A. ANT1 transfection reduced nuclear translocation of NF-κB by ANT1 over-expression. MDA-MB-231 cells were transfected with pcDNA-ANT1 and 12 hours later cells were blocked with 3% fetal bovine serum in 1% BSA/PBS for 30 min, washed with PBS three times, and incubated overnight with anti-NF-κB antibody. After washing three times, cells were incubated with anti-IgG-FITC or DAPI for 30 min. Images were obtained using a LSM510. B. ANT1 transfection reduced the nuclear translocation of NF-κB by ANT1 over-expression. Cells were transfected with pcDNA-ANT1; 12 hours later, total or nuclear extracts were prepared and Western blotted using anti-NF-κB or anti-α-tubulin antibodies. Immunoblots were visualized using an enhanced chemiluminescence detection system. C. ANT1 transfection reduced the transcriptional activity of NF-κB induced by ANT1 over-expression. Cells were co-transfected with pcDNA-ANT1 and NF-κB-luciferase reporter construct for 12 hours and then lysed. Lysate luciferase activities were determined using a luminometer.

To evaluate the effect of ANT1 over-expression on the NF-κB activity, MDA-MB-231 cells were transfected with pcDNA3.1-ANT1 for 12 hours and subjected to confocal microscopy, Western blotting, and Luciferase assay. These analyses were performed before apoptotic cell death was induced. The results obtained showed that ANT1 transfection significantly decreased NF-κB translocation to the nucleus in MDA-MB-231 cells (Fig. 2A and 2B). Luciferase assays also showed that transcriptional activity of NF-κB was inhibited in ANT1-transfected cells (Fig. 2C). These results suggested that ANT1 transfection induced inactivation of NF-κB in MDA-MB-231 cells, and thereby, subsequently caused apoptotic cell death.

### 3. Apoptotic signaling pathway was regulated byover-expression of ANT1

**Figure 3:**
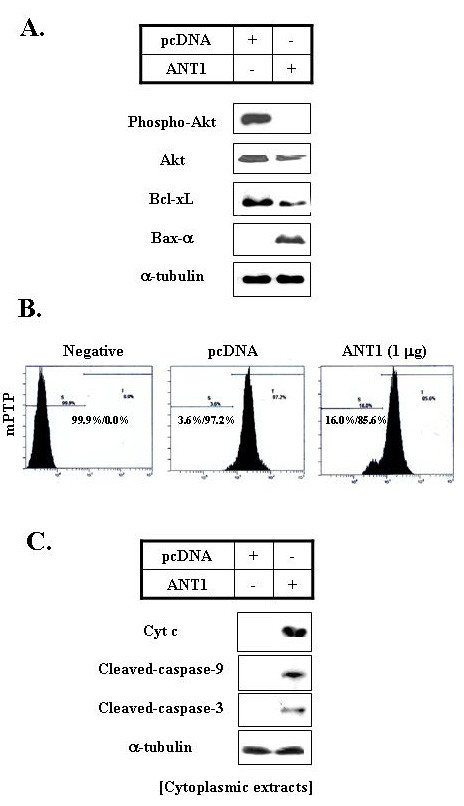
**Apoptotic signaling was regulated by ANT1 transfection**. A. ANT1 transfection induced Akt inactivation, reduced Bcl-xL expression, and Bax induction. Cells were transfected with pcDNA-ANT1 for 12 hours, and then total or nuclear extracts were prepared and Western-blotted using anti-phospho-Akt, anti-Akt, anti-Bcl-xL, anti-Bax, or anti-α-tubulin antibodies. Immunoblots were visualized by enhanced chemiluminescence. B. Mitochondrial membrane potential disruption induced by ANT1 transfection. Cells were transfected with pcDNA-ANT1 for 12 hours and then stained with DiOC_6_. Mitochondrial membrane potentials were quantified by flow cytometry. C. Caspases-9 and -3 activation induced by ANT1 transfection. Cells were transfected with pcDNA-ANT1 for 24 hours. Cytoplasmic extracts were then prepared and Western-blotted using anti-caspase-9, anti-caspase-3, or anti-α tubulin antibodies. Immunoblots were visualized by enhanced chemiluminescence.

Next, we investigated the apoptotic-signaling pathways involving in the ANT1 transfection-induced apoptosis of MDA-MB-231 cells. ANT1 transfection down-regulated Akt activation (Akt phosphorylation) and Bcl-xL protein levels, but, significantly increased Bax-α (a pro-apoptotic Bcl-2 family member) levels (Fig. 3A). Mitochondrial membrane potential (MMP) was also destroyed by ANT1 transfection, as determined by DiOC_6 _staining (Fig. 3B). Furthermore, apoptosis induced by ANT1 over-expression was accompanied by enhanced cytochrome c release, and caspases-9 and -3 activation (Fig. 3C.). All the data indicated that ANT1 transfection induced mitochondria-mediated apoptosis in MDA-MB-231 cells.

### 4. ANT1 transfection significantly inhibited tumor growth *in vivo*

**Figure 4:**
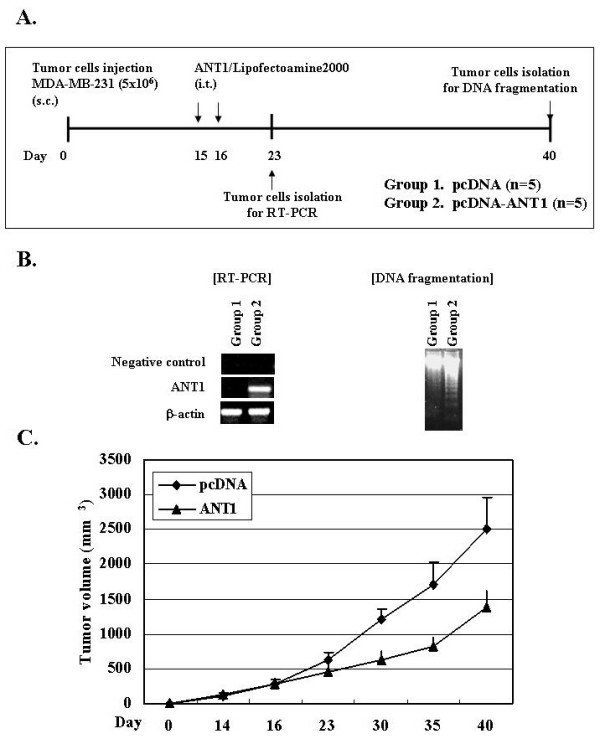
**Over-expression of ANT1 inhibits tumor growth *in vivo***. A. Animal experimental schedule is described in detail in Materials and Methods. Briefly, mice were challenged with MDA-MA-213 cells via subcutaneous injection. On day 15 and 16 after tumor cell challenge, mice were treated with pcDNA (group1) or pcDNA-ANT1 (group2) via intratumoral injection. B. On day 23, tumor cells were isolated from tumor tissues from sacrificed extra mice, and total RNA was extracted and subjected to RT-PCR to evaluate the expression of ANT1. On day 40, total DNA was also extracted and subjected to DNA fragmentation assay. C. Tumor volumes were measured weekly using a caliper for 40 days after tumor cell challenge. Tumor volumes were calculated by *m*_1_^2 ^× *m*_2 _× 0.5236, (where *m*_1 _= short tumor axis and *m*_2 _= long tumor axis).

Finally, in order to evaluate the therapeutic effect of pcDNA3.1-ANT1 *in vivo*, we established *in vivo *tumor model by subcutaneous injection of MDA-MB-231 cells into female Balb/c nude mice. Two weeks after tumor cell challenge, when the tumor volume reached approximately 100 mm^3^, mice were allocated into two groups for therapy. On day 15 and 16 after tumor cell challenge, mice were twice treated with 100 ug/100 ul pcDNA 3.1 complex with Lipofectamine 2000 in group 1 (control group, n = 5), or with 100 ug/100 ul pcDNA 3.1-ANT1 complex with Lipofectamine 2000 in group2 (therapy group, n = 5) *via *intratumoral injection. On day 23 (one week after treatment), tumor cells were isolated from tumor tissue of mice, and total RNA was extracted and subjected to RT-PCR to assess the ANT1 expression. We also performed PCR using the total RNAs as template as a negative control of PCR to exclude the plasmid contamination during the RNA extraction from tumor tissues. ANT1 was over-expressed in tumor of mice treated with pcDNA 3.1-ANT1 but not in control mice treated with pcDNA 3.1 (Fig. 4B). Tumor volumes were measured weekly throughout the experiment for 40 days (Fig. 4A). The tumor volumes of pcDNA 3.1-ANT1-treated mice were significantly smaller than those of control mice (*p *< 0.05). At the end of observation (day 40), tumor growth was inhibited up to 50% in pcDNA 3.1-ANT1-treated mice, compared with control mice (Fig. 4C). On day 40, total DNA was extracted for DNA fragmentation assay. DNA laddering was observed in tumor cells of mice treated with pcDNA 3.1-ANT1 (Fig 4B), which means that apoptotic cell death rather than cell cycle arrest might be involved in the suppression of tumor growth by pcDNA 3.1-ANT1 treatment. All of these *in vivo *results suggest that transfection of ANT1 expression vector into tumor cells could exert an anti-tumor effect *in vivo *partly by inducing apoptotic cell death.

## Discussion

Mitochondria are required for cellular bioenergetics and play a central role in determining the 'point of no return' in the apoptotic process [17]. The most commonly used cancer therapeutics eliminate tumors by inducing apoptosis [18,19]. Moreover, apoptotic signaling pathways involve mitochondrial membrane permeabilization, an event referred to as the 'central executioner' of apoptosis [20]. During this process, the mitochondrial outer and inner membranes are both permeabilized, which results in the release of soluble proteins from mitochondria, and results in activated proteases and nucleases beginning to break down the cell [21].

ANT1 is a component of the mitochondrial permeability transition pore (MPTP) and the most abundant protein in the mitochondrial inner membrane (IM; 22). MPTP is a protein complex found in mitochondrial contact sites and when it is opened acts as a non-specific channel that allows free passage of molecules < 1500 Da in size through the IM [23]. As a consequence, the MMP is reduced and the mitochondria become swollen. MPTP opening is regulated by Bcl-2 family members, such as Bax and Bcl-2, and whereas Bax activates this opening, Bcl-2 inhibits it. Moreover, during this process of MPTP opening, ANT1 interacts with Bax or Bcl-2 [24,25]. In terms of the role of NF-κB in cancer, NF-κB inhibition by ANT1 over-expression may be an effective way of suppressing tumor cell proliferation. ANT1 over-expression resulted in the recruitment of the I-κBα-NF-κB-complex into the mitochondria and a concurrent decrease in nuclear NF-κB binding activity [12]. ANT1 overexpression could be broadly applied for therapy to decrease Bcl-2 levels or inhibit NF-κB activity in various human cancers. Additionally, Bcl-2 or NF-κB over-expression occurs in many cancer types and is associated with chemoresistance. Recently, ANT1 over-expression was shown to increase the chemotherapeutic effect using all-trans retinoic acid [26].

Although ANT isoforms are of central importance with respect to MTMP, and play a major role in apoptosis, only one study has described the potential therapeutic use of ANT2 as an anti-cancer agent based on the results of siRNA knock-down studies [27]. In the present study, we adopted a gene therapy approach using ANT1 to trigger cancer cell apoptosis. ANT1 over-expression resulted in cancer cell apoptosis *in vitro *and *in vivo *through inactivation of Akt and NF-*κ*B, and mitochondria-mediated apoptotic pathway, and effectively suppressed tumor growth *in vivo*. Moreover, we achieved a synergistic effect by combination of ANT1 over-expression with cytotoxic drugs, such as a genistain or gemcitabine, for cancer therapy (data not shown).

Based on the preceding results of the present study, we suggest that ANT1 gene transfer might be an efficient strategy to suppress tumor growth and to sensitize cancer cells to chemotherapeutic agents. However, according to the reference [28], efficiency of *in vivo *gene transfer into tumors by Lipofectamine2000 is only about 2%. In our experiments, single intratumoral administration of pcDNA-ANT1 complex with Lipofectamine2000 *in vivo *could not effectively inhibit tumor growth, which was probably due to the low efficiency of gene delivery. Therefore, the development of newer gene delivery system to enhance transfection efficiency is necessary for more effective cancer gene therapy targeting ANT'1.

## Conclusion

Our results show that ANT1 transfection inactivates NF-*κ*B, induces apoptosis, and inhibits tumor growth *in vitro and in vivo*. These results may not be limited to specific cancer cell types, and thus ANT1 gene regulation of tumor-specific promoters might be used in therapy for various types of cancer. Further studies, including clinical trials, are needed to overcome low gene delivery efficiency *in vivo*.

## Competing interests

The authors declare that they have no competing interests.

## Authors' contributions

J–YJ performed most of the experiments and was responsible for producing the results, data analysis, and preparing the manuscript; YC and KCYA contributed to the conduction of additional experiments; Y–KJ was responsible for analysis of data and writing of the paper; C–WK contributed to the design of the project, data analysis and writing of the paper. All authors reviewed and agreed the final manuscript.

## Pre-publication history

The pre-publication history for this paper can be accessed here:


